# A survey of model compression techniques: past, present, and future

**DOI:** 10.3389/frobt.2025.1518965

**Published:** 2025-03-20

**Authors:** Defu Liu, Yixiao Zhu, Zhe Liu, Yi Liu, Changlin Han, Jinkai Tian, Ruihao Li, Wei Yi

**Affiliations:** Intelligent Game and Decision Lab (IGDL), Beijing, China

**Keywords:** model compression, deep neural networks, large language model, pruning, quantization, low-rank decomposition, knowledge distillation

## Abstract

The exceptional performance of general-purpose large models has driven various industries to focus on developing domain-specific models. However, large models are not only time-consuming and labor-intensive during the training phase but also have very high hardware requirements during the inference phase, such as large memory and high computational power. These requirements pose considerable challenges for the practical deployment of large models. As these challenges intensify, model compression has become a vital research focus to address these limitations. This paper presents a comprehensive review of the evolution of model compression techniques, from their inception to future directions. To meet the urgent demand for efficient deployment, we delve into several compression methods—such as quantization, pruning, low-rank decomposition, and knowledge distillation—emphasizing their fundamental principles, recent advancements, and innovative strategies. By offering insights into the latest developments and their implications for practical applications, this review serves as a valuable technical resource for researchers and practitioners, providing a range of strategies for model deployment and laying the groundwork for future advancements in model compression.

## 1 Introduction

Deep learning has rapidly developed since 2012, demonstrating strong capabilities in representation learning and achieving remarkable success across various fields. Notably, the accomplishments of deep networks on the ImageNet benchmark Jia et al. (2009) have significantly propelled research on deep networks and their applications. The effectiveness of deep learning lies in its ability to transform raw data into abstract representations, facilitating the discovery, learning, and automatic representation of data features. By employing a hierarchical structure, deep learning models learn feature representations at different levels, allowing them to progressively capture both simple concepts and complex abstract features. Research indicates that in deep neural networks, layers near the input learn lower-level features, while layers closer to the output capture more complex concepts (Yann et al., 2015). This phenomenon is likely due to the deeper layers having a larger receptive field.

The success of deep learning models can be attributed to the availability of large datasets, increased computational power, and advancements in model architecture. With the maturity of internet applications, various fields have accumulated substantial amounts of data, while rapid advancements in chip technology have significantly enhanced computational capabilities. Deep learning models have become the preferred choice across numerous applications due to their superior performance. Research suggests that increasing the depth of neural networks generally improves model performance (Li et al., 2020). However, deeper models also require significantly more computational resources and memory. For example, AlexNet, introduced with a parameter count of 240 MB, was soon followed by VGG-Net, which required over 500 MB of parameters. The limited memory and computational power of many edge devices severely constrain the deployment of deep models in edge computing environments (Wu et al., 2016; Han et al., 2016). Despite their large number of learnable parameters, these models exhibit considerable redundancy Denton et al. (2014), resulting in high computational costs and substantial storage demands. For instance, the VGG-16 model requires over 30 billion floating-point operations (FLOPs) to classify a single image (Zhou et al., 2017). Therefore, optimizing models to reduce computational and storage requirements, while maintaining performance, is crucial for expanding the applicability of deep learning models to mobile devices, embedded systems, and real-time applications (Zhou et al., 2017).

The success of the GPT-3 model has significantly advanced the application of large models across various fields, particularly propelling the development of models in specific vertical domains. However, large models have reached an unprecedented scale with hundreds of millions of parameters, making the training of such models a task that only a few teams worldwide can accomplish. Even deploying these models requires extremely high hardware support. For instance, the Llama2 model, which has 130 billion parameters (Touvron et al., 2023), requires 260 GB of memory even when using half-precision float16 (2 bytes per parameter). The successful application of large models has significantly increased the tension between algorithmic demands and low-power hardware, underscoring the urgent need for more efficient model compression algorithms to address the limitations of computational power and memory capacity in hardware.

In summary, although over-parameterized models typically achieve strong performance, deploying them directly on edge devices presents considerable challenges due to their substantial hardware demands. These models often have a large number of parameters, leading to high memory usage, significant inference latency, and increased computational power consumption. Consequently, these factors greatly limit the feasibility of deploying deep models on edge devices.

To address the above challenges, model compression techniques have become a popular research direction for deploying large/deep models on edge devices with limited computational power, aiming to minimize performance loss while efficiently deploying “large” models (with high parameter counts) onto “small” devices (with low computational resources).

### 1.1 Motivation and contributions

Recent research indicates that both fully connected and convolutional neural networks possess a significant number of redundant parameters when trained on limited datasets (Frankle and Carbin, 2019; Frankle et al., 2020; Chen et al., 2020; Da Cunha et al., 2022). Although the large number of redundant parameters in models significantly contributes to their learning and generalization capabilities, they also present two major challenges during deployment: the limited computational power and memory capacity of edge devices. Constrained by size and power consumption, edge devices have limited memory and computational resources. Running inference with the original model not only consumes a large amount of memory but also results in high power consumption, long inference times, and slow response speeds. Even when disregarding power consumption and inference speed, the memory limitations alone can make it challenging to deploy many deep learning models directly on edge devices. To facilitate the deployment of deep models on edge devices, increase inference speed, and reduce power consumption, it is essential to compress the models by eliminating redundant parameters and reducing their overall size. This compression decreases memory requirements and computational load during inference, thereby achieving faster inference speeds.

This paper conducts an extensive literature review on model compression techniques, focusing on providing a deeper explanation of different types of compression methods. The research categorizes these techniques into four domains: model pruning, model distillation, low-rank decomposition, and quantization. It emphasizes the compression methods and their underlying theories, offering a detailed analysis of the performance of various compression approaches. Furthermore, it explores several promising future directions, such as pruning algorithms that do not require fine-tuning and fully quantized model compression techniques. Ultimately, this paper aims to present a broad overview of model compression technologies and provide valuable insights for selecting appropriate techniques for compressing deep models.

## 2 Related literature

This paper broadly classifies model compression techniques into three stages: the period prior to the emergence of deep models is referred to as the era of shallow networks; the period following the advent of deep learning but before the emergence of large models is identified as the era of deep models; and the period after the rise of large models is termed the era of large models. Due to constraints on the scope of this section, we provide an overview of representative works from these three stages.

### 2.1 Shallow network era (before 2012)

The initial model compression techniques can be traced back to the early 1980s. At that time, compression primarily aimed to reduce computational complexity by eliminating non-essential network parameters, a technique known as pruning. Conceptualized during the early 1980s and 1990s, pruning was applicable to any part of a deep neural network (Mozer and Smolensky, 1988; Hanson and Pratt, 1988; Romaniuk, 1993; Weigend et al., 1991a; Weigend et al., 1991b; Hassibi and Stork, 1992; Reed, 1993). The pioneering works of LeCun et al. (1989), who proposed Optimal Brain Damage (OBD), and Hassibi and Stork (1992), who introduced Optimal Brain Surgeon (OBS), demonstrated that unimportant weights could be removed from trained networks without significantly affecting performance. Later, to identify the minimal network enabling a robot to perform a specific behavior without predefining the required sensors, Becerra et al. (2002) proposed Gaussian Synapse Networks. This structure proved highly efficient for developing behavior-based controllers by utilizing evolutionary techniques to prune the network rather than retraining it—a particularly insightful approach given the computational resource limitations at the time. These methods laid the foundation for model compression and profoundly influenced subsequent developments. However, since both techniques rely on calculating and ranking second-order derivatives to identify and remove unimportant weights in an iterative process, they are more suited to shallow networks (typically with fewer than three layers). For modern deep models, known for their complexity, the retraining costs are too high for direct application. Nevertheless, the principles behind pruning and optimization continue to influence model compression today. For example, modern quantization methods, such as AdaRound Nagel et al. (2020), incorporate second-order derivative information to optimize quantization functions.

In summary, the primary goal of pruning algorithms is to extract a sub-network with fewer parameters without compromising accuracy. The pruned network, as a smaller version of the original, can represent the model with reduced size or parameter count (Denil et al., 2013). Over-parameterized networks can thus be effectively compressed while maintaining strong generalization performance (Frankle and Carbin, 2019; Sun et al., 2022; Arora et al., 2018). Therefore, a key direction in pruning research focuses on designing methods that reduce computational costs or increase compression rates, while selecting optimal network structures without significantly degrading prediction accuracy.

### 2.2 Deep model era (2012–2022)

Early pruning work laid the groundwork for modern compression techniques, with subsequent research extending these ideas and developing advanced compression methods suited to deep models.

Deep learning, synonymous with deep neural networks (typically more than three layers), integrates feature learning and representation into a single framework through deep architectures. These models have achieved exceptional performance in various tasks, particularly convolutional networks, which excel in machine vision tasks such as image recognition, object detection, and semantic segmentation. In 2012, AlexNet Krizhevsky et al. (2012) reduced the error rate in the ImageNet image recognition competition by approximately 10 percentage points, winning the championship and demonstrating the powerful capabilities of deep models for the first time. It is considered the first convolutional network to achieve a breakthrough in large-scale image recognition. At that time, the model size was 240 MB, which far exceeded the memory capacity of many on-chip systems, posing new challenges for deployment. During this period, the prevailing belief was that the deeper the network, the better its generalization performance. In Simonyan and Zisserman (2015), the authors introduced VGGNet, a classic example of deep convolutional networks. By this time, the parameters of VGG16 had grown to 528 MB, presenting an even greater challenge for hardware. As a result, reducing the size of deep models without significantly sacrificing accuracy became one of the major research focuses.

In response to the growing size of deep models, researchers achieved several landmark breakthroughs in model compression. For instance, in Han et al. (2016), the authors introduced the concept of deep compression, combining pruning, quantization during training, and Huffman coding, significantly reducing memory usage in deep neural networks without substantial performance loss. The core innovation, training quantization, groups weight parameters into clusters, each sharing a floating-point value, thus dramatically reducing memory overhead. This concept paved the way for subsequent quantization methods. Zhou et al. (2017) further refined this approach by allowing a portion of the weights to retain full precision while quantizing others, reducing quantization errors. In their study Krishnamoorthi, 2018), the authors found that applying conventional quantization methods to MobileNetV2 (Sandler et al., 2019) led to a drastic performance drop from 70.9% to 0.1% on ImageNet (Jia et al., 2009). Building on this finding, in Nagel et al. (2019), the authors observed that many of the model’s channels were quantized to zero, obscuring the differences between channels. In response, they proposed a data-free quantization method for deep neural networks that balances the weight range based on the scale-invariance property of activation functions. This approach eliminates the need for fine-tuning or hyperparameter selection, achieving performance comparable to the original model across common computer vision architectures and tasks.

In academia, some researchers pursue extreme compression rates, focusing on theoretical rigor and achieving the lowest possible bit widths. In Hubara et al. (2016), the authors proposed binary neural networks, a radical compression method that uses only +1 and −1 to represent all values, including weights and activations, achieving fully quantized inference. Without degrading accuracy, this drastically reduced parameter size and increased inference speed. Simultaneously, in Courbariaux et al. (2015), the authors introduced BinaryConnect, which trains binary weights (1 or −1 ) during forward and backward propagation, compressing parameters to an extreme 1-bit format while retaining gradient accuracy. Like dropout solutions, BinaryConnect acts as a regularizer, achieving near state-of-the-art results on datasets like MNIST, CIFAR-10, and SVHN. Improved methods such as XNOR-Net (Rastegari et al., 2016), DoReFa-Net (Zhou S. et al., 2016), and Ternary Weight Networks Li et al. (2016) followed, building on this extreme quantization concept. In Zhao et al. (2021), the authors propose a novel Bayesian Optimized compact 1-bit CNNs (BONNs) model, leveraging the advantages of Bayesian learning to significantly enhance the performance of 1-bit CNNs. BONNs integrate the prior distributions of full-precision kernels, features, and filters into a Bayesian framework to construct 1-bit CNNs in a comprehensive end-to-end manner. The proposed Bayesian learning algorithms are well-structured, optimizing the network across different kernels, features, and filters, thereby improving both the compactness and capacity of 1-bit CNNs. Additionally, they introduce a Bayesian learning-based pruning method, which significantly boosts model efficiency while maintaining competitive performance. This makes the method highly applicable across various practical scenarios. Extensive experiments on datasets such as ImageNet, CIFAR, and LFW demonstrate that BONNs outperform a variety of state-of-the-art 1-bit CNN models in classification tasks, while also exhibiting strong generalization performance in object detection.

However, industry applications demand solutions that can be practically deployed on hardware while maintaining accuracy. Current 2-bit or 3-bit quantization methods often require specialized hardware, limiting their widespread deployment. In response, companies like Google proposed a more general quantization standard (Jacob et al., 2018), which has been implemented in frameworks such as TensorFlow Lite (TFLite), PyTorch, and ONNX. Additionally, the neural-compressor, an open-source Python library released by Google, supports various popular model compression techniques. In this context, 8-bit quantization has become crucial for efficient inference on modern hardware, offering a balance between performance and compatibility. Many trained FP32 models can be quantized to INT8 with minimal loss in accuracy. Some of the post-training static quantization results are listed in Table 1.

**TABLE 1:** Post-training static quantization results across different models.

Model	Domain	Approach	Example	Accuracy		
				INT8	FP32	Ratio
ResNet50 v1.0	Image Recognition	Post-Training Static Quantization	pb	74.11%	74.27%	−0.22%
ResNet50 v1.5	Image Recognition	Post-Training Static Quantization	pb/keras	76.25%	76.46%	−0.28%
MobileNet V3	Image Recognition	Post-Training Static Quantization	pb	76.72%	76.75%	−0.03%
Inception ResNet V2	Image Recognition	Post-Training Static Quantization	pb/keras	80.25%	80.40%	−0.18%
ViT	Image Recognition	Post-Training Static Quantization	pb	81.39%	81.92%	−0.64%
DenseNet161	Image Recognition	Post-Training Static Quantization	pb	76.29%	76.29%	0.00%
BERT large SQuAD (Model Zoo)	Natural Language Processing	Post-Training Static Quantization	pb	92.36%	92.98%	−0.67%
BERT large SQuAD	Natural Language Processing	Post-Training Static Quantization	pb	92.44%	92.99%	−0.58%
Transformer LT	Natural Language Processing	Post-Training Static Quantization	pb	25.82%	25.86%	−0.15%
Transformer LT MLPerf	Natural Language Processing	Post-Training Static Quantization	pb	27.13%	27.17%	−0.13%
Mask R-CNN Inception V2	Object Detection	Post-Training Static Quantization	pb/ckpt	28.46%	28.73%	−0.91%
YOLOv3	Object Detection	Post-Training Static Quantization	pb	83.28%	82.35%	1.12%

These landmark studies have played a critical role in model compression, significantly reducing the size and computational complexity of deep neural networks and inspiring further development of efficient architectures and compression techniques.

### 2.3 Large model era (2022-now)

With the introduction of BERT (Devlin et al., 2018) and GPT-1/2 (Radford and Narasimhan, 2018; Radford et al., 2019), and especially the success of large models like GPT-3/4 (Brown, 2020; OpenAI et al., 2024), ChatGPT, and Claude (Caruccio et al., 2024), model compression faces new challenges: large models prioritize generalization and versatility over performance on specific tasks. Besides, due to their massive size, even inference for large, highly-accurate LLMs may require multiple performant GPUs, which limits the usability of such models. While there is emerging work on relieving this pressure via model compression, the applicability and performance of existing compression techniques is limited by the scale and complexity of LLMs. Research on compression techniques for large language models (LLMs) has expanded significantly to address the growing demands for efficient deployment on various hardware platforms. These techniques aim to reduce the computational cost and memory footprint of LLMs while retaining their performance. In this situation, some of the milestone methods have been proposed for LLMs compression. In Kurtic et al. (2022), the authors tackle the challenge of sparsifying BERT models, a fundamental component in natural language processing. They introduce the Optimal BERT Surgeon (oBERT), an efficient and precise pruning method based on approximate second-order information, which achieves state-of-the-art compression results in both pre-training and fine-tuning stages of language tasks. Specifically, oBERT enhances existing second-order pruning techniques by enabling the pruning of weight blocks and is the first approach of its kind scalable to BERT-sized models. Furthermore, the authors explore compounding compression techniques to create highly compressed yet accurate models suitable for deployment on edge devices. These models set new benchmarks in sparse BERT model performance across various metrics, including model size, inference speed, and task accuracy. For example, compared to the base dense BERT model, oBERT achieves a 10x reduction in model size with less than 1% accuracy drop, a 10x CPU-inference speedup with under 2% accuracy drop, and a 29x CPU-inference speedup with less than 7.5% accuracy drop. In Frantar et al. (2023), the authors propose OPTQ, a novel one-shot weight quantization method based on approximate second-order information that is both highly accurate and efficient. Specifically, OPTQ can quantize GPT models with 175 billion parameters in roughly four GPU hours, reducing the bitwidth to 3 or 4 bits per weight with minimal accuracy loss relative to the uncompressed baseline. This method represents the first successful execution of a 175 billion-parameter model on a single GPU for generative inference. Moreover, the method achieves reasonable accuracy even under extreme quantization conditions, such as 2-bit or ternary quantization. Experimental results show that these improvements can lead to significant end-to-end inference speedups over FP16, achieving approximately 3.25x acceleration when using high-end GPUs, like the A100. In Cheng et al. (2023), the authors introduce TEQ, a trainable equivalent transformation designed to maintain FP32 precision in model outputs while leveraging the benefits of low-precision quantization, particularly 3 and 4-bit weight-only quantization. The training process is efficient, requiring just 1 K steps and less than 0.1% of the original model’s trainable parameters. Additionally, the transformation introduces no computational overhead during inference. The results are comparable to state-of-the-art (SOTA) methods on standard large language models (LLMs). Moreover, this approach can be combined with other techniques to achieve even better performance. In Lin et al. (2024), the authors observed that not all weight parameters are equally important. Based on this insight, AWQ uses activation values from calibration data to select the top 1% of important parameters, which retain full precision, while the remaining parameters are quantized to 4-bit, achieving nearly 8x compression without compromising large model performance. Moreover, in Xu et al. (2024a), the authors further pushed the boundaries by using shared parameters to achieve extreme 1-bit quantization, where parameter tensors consist solely of 1, −1 . During inference, the original weights are reconstructed through dot products of shared parameter vectors, reducing memory usage by 90% without significantly degrading performance.

## 3 Model compression methods

**FIGURE 1:**
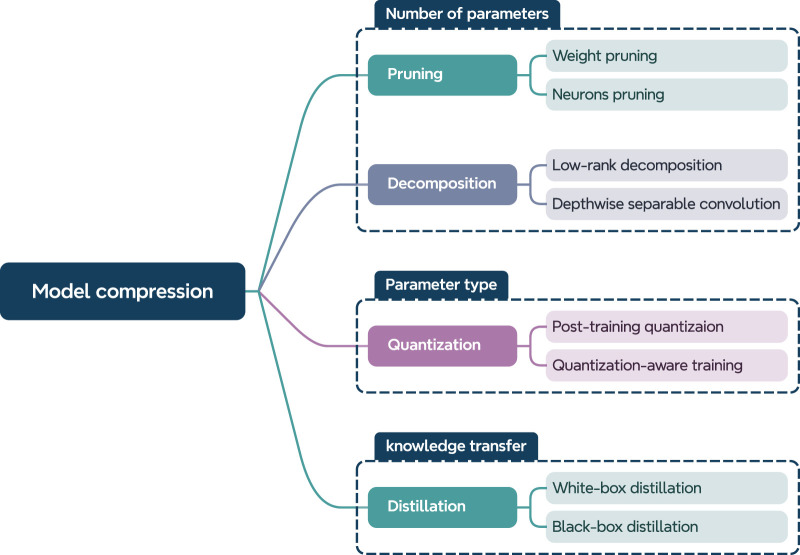
A taxonomy of model compression based on the focus of each method.

The goal of model compression is to significantly reduce the number of parameters, improve inference speed, and lower response latency, all while maintaining the model’s generalization performance. One intuitive approach is to reduce the number of parameters in deep models, which can be done in two ways: First, redundant parameters in the network can be eliminated, a method known as pruning. Second, large parameter tensors can be decomposed into smaller tensors with fewer parameters, a technique called low-rank decomposition. Another strategy involves reducing the memory footprint of each parameter without decreasing the total number of parameters, referred to as quantization. Furthermore, from a knowledge transfer perspective, knowledge distillation is also applied in model compression. Specifically, a smaller network is trained to learn from the original model, aiming to replicate its performance; this technique is known as model distillation. As described above, the key compression methods—quantization, pruning, low-rank decomposition, and knowledge distillation—are illustrated in Figure 1. This paper classifies model compression techniques into four general categories: pruning, low-rank decomposition, quantization, and distillation. Each method is discussed in detail in the following sections.

### 3.1 Quantization methods

Quantization involves reducing the precision of model weights and activations from high-precision formats (e.g., FP32) to lower-precision formats (e.g., INT8 or lower), which reduces memory usage and accelerates inference. Mathematically, quantization maps continuous floating-point numbers to discrete fixed-point numbers. For instance, model parameters can be converted from 32-bit floating-point (FP32) to 8-bit integer (INT8), or even to extremely low-bit formats like 2-bit or 3-bit fixed-point data types. Some approaches have even employed 1-bit quantization to achieve maximal compression. A float32 value requires 4 bytes of memory, while INT8 only needs 1 byte, and INT4 just half a byte. This form of quantization significantly reduces the model’s memory footprint, helping mitigate the constraints on deep model deployment in edge devices, particularly for large models. Moreover, since fixed-point operations can be performed via bit-shifting, converting floating-point values to fixed-point not only accelerates memory access by several times but also makes fixed-point operations more amenable to hardware acceleration. This further reduces the computational demands on hardware and accelerates the deployment of deep models on edge devices.

#### 3.1.1 Quantization theory

**FIGURE 2:**
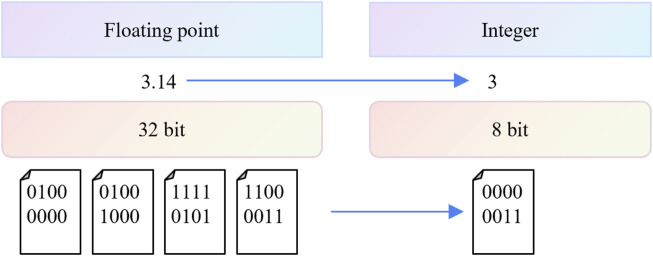
The process of model quantization.

Quantization is essentially a mapping from floating-point numbers to fixed-point numbers, while dequantization reverses this by mapping fixed-point numbers back to floating-point numbers. For example, during image preprocessing, it is common to scale an image with unsigned 8-bit integer values ranging from 0 to 255 into a tensor with 32-bit floating-point values ranging from 0.0 to 1.0; this process is called dequantization. Similarly, converting model parameters from a range of 0.0–1.0 into unsigned 8-bit integers with values between 0 and 255 is known as quantization. As shown in Figure 2, the core principle of model quantization is to represent model parameters using fewer bits. While dequantization generally does not cause information loss, quantization often leads to a reduction in precision. This occurs because float32 has a wider range and greater precision than uint8, meaning many values cannot be exactly represented in uint8 and must be rounded. The difference between a quantized model and a full-precision model arises from rounding and clipping during the quantization process. Essentially, quantization converts floating-point numbers into fixed-point numbers, discretizing the parameter values.

The simplest form of quantization divides a continuous range of real values into a finite number of intervals (e.g., d-bit integers create $2^{d}$ intervals), and all real values within the same interval are mapped to the same integer. This process can be described by the following Equation 1:

$$q=\text{round}\left(\frac{r}{S}+Z\right)$$ (1)

where r represents the floating-point value, S is the scaling factor that defines the ratio between real numbers and integers, and Z is the zero point, corresponding to the integer representation of zero in the real number range. The rounding function round(⋅) adjusts the values to the nearest integer. The scaling factor S and zero point Z are calculated as shown in Equations 2, 3:

$$S=\frac{r_{\max}-r_{\min}}{q_{\max}-q_{\min}}$$ (2)

$$Z=\text{round}\left(q_{\max}-\frac{r_{\max}}{S}\right)$$ (3)

where $r_{\max}$ and $r_{\min}$ are the maximum and minimum real values, and $q_{\max}$ and $q_{\min}$ are the maximum and minimum values in the quantized range. It is important to note that the zero point Z in fixed-point representation corresponds exactly to zero in the real number range without any loss of precision after quantization.

In quantized models, two key concepts are the value range and clipping. In uniform quantization, an important factor is defining the value range [α,β] , where values below α are clipped to α , and values above β are clipped to β . This value range directly impacts the scaling factor S in uniform quantization.

$$S=\frac{\beta -\alpha }{2^{b}-1}$$ (4)

In general, a wider value range reduces the likelihood of outliers in the input data being clipped. However, this comes at the cost of more data points being mapped to the same fixed-point value, which can result in significant information loss and a rapid degradation in the performance of the quantized model. As shown in Equation 4, this is the trade-off associated with using a larger scaling factor.

To optimize quantization performance, the value range typically needs to be calibrated based on the training data. This calibration is not a fine-tuning or retraining of the model, but rather a selection process to determine the appropriate weight range. Common calibration methods include using the maximum and minimum values, the absolute maximum value, and minimizing the quantization error between floating-point and integer representations (KL-divergence).

#### 3.1.2 Overview of quantization methods

**FIGURE 3:**
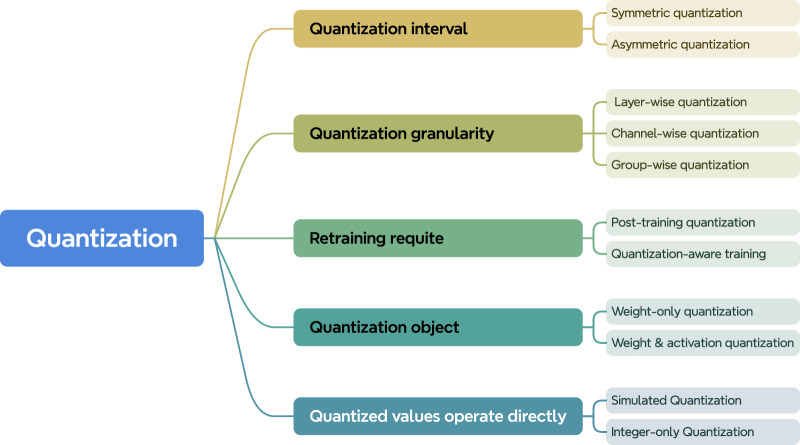
A taxonomy of quantization based on the focus of each method.

Here, we briefly introduce some concepts related to quantization methods. Although quantization fundamentally involves mapping model parameters from floating-point to integer values, quantization methods can be categorized in various ways depending on the specific approach. As shown in Figure 3, quantization methods can be classified according to factors such as the partitioning of quantization intervals, the granularity of quantization, whether retraining is required, the target of quantization, and the type of operations executed during inference. Below, the main quantization methods are defined and explained.

1. Symmetric and Asymmetric Quantization: Quantization can be classified into symmetric and asymmetric quantization based on whether the quantization interval is symmetric. In symmetric quantization, the condition α+β=0 holds, whereas if this condition is not satisfied, it is referred to as asymmetric quantization.
2. Quantization Granularity: Based on the granularity of application, quantization methods are divided into layer-wise, channel-wise, and group-wise quantization. Typically, finer granularity leads to better quantization results but requires more storage for parameters (e.g., scaling factors) and increases computational cost. Therefore, many methods balance the model’s performance and computational overhead by adjusting the granularity of quantization.
3. Post-Training Quantization (PTQ) and Quantization-Aware Training (QAT): Depending on whether the model requires retraining after quantization, methods are divided into PTQ and QAT. QAT involves retraining or fine-tuning the model after quantization to reduce quantization errors, while PTQ applies quantization without retraining, though it requires a calibration set to correct deviations caused by quantization.

Given the limited range of fixed-point numbers, quantization inevitably introduces quantization errors, which can cause the model to deviate from the optimal point achieved with floating-point precision. To mitigate this, QAT simulates the quantization process during retraining or fine-tunes the model to help it converge to a better point. However, since QAT requires retraining, it can be prohibitively expensive for large models with billions of parameters (LLMs). To reduce the cost of retraining, PTQ uses non-training methods (e.g., data calibration) to achieve similar performance to the original model without additional computation. Since PTQ does not involve retraining, it is generally less effective than QAT, which fine-tunes the model by adjusting quantization parameters during retraining. Notably, PTQ can still achieve nearly the same performance as the original model when quantizing weights to 4 bits, as seen in methods like DFQ (Nagel et al., 2019), GPTQ/OPTQ (Frantar et al., 2023), and AWQ (Lin et al., 2024).

4. Weight-only Quantization and Weight and Activation Quantization: These methods differ based on whether only the weights or both weights and activations are quantized. Weight-only quantization reduces the precision of the weight parameters without affecting the activations (neuron outputs), thus preserving the original precision of the network outputs while compressing the model using fewer bits, leading to lower memory requirements.

Weight and activation quantization methods reduce both the weight parameters and activations, significantly lowering memory and computational demands. However, compressing activations can result in a greater loss of feature information, increasing quantization error and potentially degrading the model’s generalization performance (Wei et al., 2022; Xiao et al., 2023). Moreover, with the advancement of hardware, the speed of model inference has significantly improved, alleviating computational bottlenecks. As a result, the main challenge in quantizing large language models (LLMs) has shifted toward memory limitations.

5. Simulated Quantization and Integer-only Quantization: These methods differ based on whether dequantization is required during inference. Simulated quantization, also known as fake quantization, quantizes only the weight parameters, using low-precision types (e.g., INT4) and dequantizing back to high-precision types (e.g., FP16) during inference. In contrast, integer-only quantization performs all inference calculations using integer operations, benefiting from hardware acceleration for low-bit operations. Weight-only quantization is classified as simulated quantization, while weight + activation quantization is classified as integer-only quantization.

While quantization methods can be categorized in detail, many approaches often overlap across multiple classifications. For example, the AWQ method falls under PTQ, channel-wise quantization, and symmetric quantization simultaneously. Therefore, in conclusion, although various quantization methods exist, PTQ and QAT remain the most prevalent approaches for model quantization. This paper primarily focuses on the application of PTQ and QAT techniques for large-scale models.

#### 3.1.3 Post-training quantization

With the rapid advancement of large language models, research on post-training quantization (PTQ) methods has significantly increased. This rise in interest is primarily due to PTQ methods not requiring the resource-intensive retraining process of large models, making them a feasible research direction for most researchers. Post-training quantization methods can be categorized based on the target of quantization into two types: weight-only quantization and weight and activation quantization. Below, we will introduce post-training quantization methods according to these different quantization targets.

##### 3.1.3.1 Weight-only quantization

Early PTQ methods focused on minimizing the error between the original model’s weight matrix W and the quantized weight matrix Q(W) , formulated as (Equation 5):

$$\arg\underset{Q}{\min}\|W-Q\left(W\right)\|$$ (5)

Some studies adapted rounding techniques with minor modifications to directly apply them to quantizing large models (Nagel et al., 2020; Yao et al., 2023; Zeng et al., 2023; Yao et al., 2022; Kim et al., 2023). In Nagel et al. (2019), the authors proposed a data-free quantization (DFQ) method for deep neural networks, which eliminates the need for fine-tuning or hyperparameter selection. This method achieved near-original performance across standard computer vision architectures and tasks. The use of 8-bit fixed-point quantization is critical for efficient inference on modern deep learning hardware. In Yao et al. (2022), the authors introduced the ZeroQuant method for the OPT and BLOOM models, which quantized model parameters to 8-bit using symmetric row-wise quantization, while preserving the precision of activation values in FP16 or FP32. This approach enabled parameter compression without sacrificing model performance. However, when the model parameters were quantized to 4-bit using this method, a sharp decline in performance was observed. Subsequently, in Yao et al. (2023), the authors proposed ZeroQuant-v2, which applied Low-Rank Compensation (LoRC) to mitigate the quantization error E between the original weight matrix W and the quantized weight matrix $\hat{W}$ , by using storage-efficient low-rank matrices. This allowed $E+\hat{W}$ to better approximate W . Besides, in Zeng et al. (2023), the authors found that the GLM-130B model could directly quantize its parameters to 4-bit without incurring performance loss when using a row-wise quantization approach. Their analysis further revealed that the main reason for the success of this quantization in GLM-130B, as opposed to the OPT and BLOOM models, lies in the more uniform weight distribution of GLM-130B, which results in fewer values falling outside the quantization range, thus reducing the quantization error.

However, due to the highly nonlinear nature of neural networks, even when the weight space distance is sufficiently small, it does not necessarily ensure a small error between the outputs of the original model and the quantized model. Thus, given a small representative subset C , referred to as a calibration set, one can optimize the difference between the activations of the original layer and the quantized layer to reduce the quantization error. These methods typically optimize the rounding process of the quantization function by using second-order derivatives of the loss function to decide whether to round up or down, thereby achieving more precise quantization. Mathematically, this is represented as Equation (6):

$$\arg\underset{Q}{\min}\|XW-XQ\left(W\right)\|,X\in C$$ (6)

For instance, in Nagel et al. (2020), the authors proposed a post-training weight optimization mechanism, the AdaRound quantization method, which adapts better to the data and task-specific losses. This method is both efficient and does not require network fine-tuning, relying only on a small amount of unlabelled data for calibration. The authors theoretically analyzed the rounding problem in quantizing trained neural networks. By approximating the target loss using a series expansion (likely the Taylor expansion) of the original value, they transformed the rounding of floating-point data matrices during quantization into a smooth quadratic binary optimization problem. Without fine-tuning, AdaRound successfully quantized the weights of the ResNet18 and ResNet50 networks to 4-bit with only a 1% accuracy loss. Later, Frantar et al. (2023) introduced OPTQ, a one-shot weight quantization method that utilizes second-order information to achieve high accuracy while improving inference efficiency. Specifically, OPTQ was able to quantize the GPT model, containing 175 billion parameters, reducing the precision of each parameter to 3 or 4 bits with minimal accuracy loss compared to the uncompressed original model. In general, OPTQ more than doubled the compression efficiency compared to previous one-shot quantization methods and was the first method to enable inference of the compressed GPT-175B model on a single GPU.

##### 3.1.3.2 Weight + activation quantization

Similar to weight-only quantization methods, joint weight and activation quantization can also employ basic uniform quantization techniques (Yao et al., 2022; Dettmers et al., 2022; Yuan et al., 2023), though it requires particular consideration of outliers in activations. Dettmers et al. (2022) noted that as large language models (LLMs) grow in size, extreme outliers in activations become more prevalent and exhibit consistent, systematic patterns. Building on this observation, the authors proposed the LLM.int8 pseudo-quantization algorithm for the feedforward and attention projection layers of transformer models. This approach reduces the memory requirements by half while preserving near-original accuracy. Specifically, the method begins by applying a form of corrected quantization, where each inner product within matrix activations undergoes individual normalization, accompanied by parametric quantization and sparsification. To manage outliers, a novel mixed-precision decomposition scheme is introduced, isolating the outlier dimensions into a 16-bit matrix, while over 99.90% of values are handled using 8-bit corrected quantization. The LLM.int8 method enables quantization of models with 175 billion parameters from 16/32-bit down to Int8, without compromising performance. This makes it feasible to deploy models like OPT-175B and BLOOM on a single server equipped with consumer-grade GPUs.

RPTQ Yuan et al. (2023) extends this approach by further isolating outliers into an additional matrix and reorganizing activation dimensions $X\in R^{N\times D_{\text{in}}}$ based on their minimum and maximum values. The key idea is to cluster dimensions with significant outliers into the same group and reorder them layer by layer. It is noteworthy that the statistical characteristics of each activation dimension are measured using a calibration set, allowing the reordering of outlier dimensions to be pre-determined before inference. To minimize latency, RPTQ integrates the reordering process with other operations: 1) it combines the reordering with the LayerNorm operation, eliminating unnecessary data movement and adjustments, and 2) it reorders the weight matrix columns to achieve uniform dimension alignment in the model’s output.

Recently, low-bit formats (such as FP4 and FP8) have emerged as leading alternatives for LLM quantization (Zhang et al., 2024; Wu et al., 2023). The FP8 format, supported by prominent hardware vendors like NVIDIA, offers a wider data range and greater precision, though it incurs higher hardware costs. Intuitively, low-bit floating-point formats can be seen as a special case of non-uniform quantization, providing an extended range and finer granularity. These properties help mitigate the issue of outliers in activations. Both kernel-based mixed-precision quantization [Mixture-of-Formats Quantization, MoFQ (Zhang et al., 2024)] and ZeroQuant-FP (Wu et al., 2023) have demonstrated that for activation quantization, FP8 consistently outperforms INT8.

#### 3.1.4 Quantization-aware training

Quantization-Aware Training (QAT) is a technique used to mitigate the performance degradation caused by quantization by retraining a quantized model. As noted in earlier sections, QAT has seen significant success in models preceding large language models (LLMs). However, this approach typically requires retraining the full set of model parameters, which is prohibitively expensive for LLMs. As a result, there have been efforts to combine quantization with parameter-efficient training methods to substantially reduce the cost of applying QAT to LLMs.

In the context of LLMs, QAT can be applied by retraining models on smaller datasets without compromising their emergent capabilities. LLM-QAT (Dettmers et al., 2024) directly applies the basic QAT framework (Zhou et al., 2017) for model quantization. To address the challenges of this approach, LLM-QAT introduces a data-free distillation technique, where the original model generates data and the quantized LLM is trained on this generated data to align its output distribution with the original model. Additionally, LLM-QAT incorporates key-value cache quantization and QAT, which are memory-intensive during long-sequence generation. To further reduce the overhead caused by discrepancies in precision between weights and activations, Wu et al. (2023) proposed a layer-wise knowledge distillation approach called the ZeroQuant-FP method, which progressively quantizes the weights of the original LLM using it as a teacher model.

QLoRA Dettmers et al. (2024) reduces LLM weight precision to a 4-bit data type and backpropagates gradients to Low Rank Adapters (LoRA), achieving 99.3% of the original model’s performance. Several key innovations in QLoRA enable memory savings without performance loss: (1) 4-bit NormalFloat (NF4), a novel data type optimized for normally distributed weights; (2) double quantization, which compresses the model further by quantizing the quantization parameters, albeit with some trade-offs in computation speed; and (3) the use of a Page Optimizer to manage memory. By leveraging these techniques, QLoRA is able to fine-tune LLMs with up to 65 billion parameters efficiently on a GPU with just 30 GB of memory.

Building on QLoRA, QA-LoRA Xu et al. (2024b) introduces grouped quantization. The authors argue that the number of quantized parameters in QLoRA is significantly smaller than that of LoRA parameters, leading to an imbalance between the quantization process and low-rank adaptation. Grouped operations help address this by increasing the number of quantized parameters and reducing the adaptive parameters. Additionally, LoftQ Li et al. (2024) identifies that the zero initialization of LoRA matrices in QLoRA is ineffective for downstream tasks. To improve this, LoftQ proposes initializing LoRA matrices using the singular value decomposition (SVD) of the difference between the original and quantized weights, alternating between quantization and SVD to better approximate the original weights.

### 3.2 Pruning methods

**FIGURE 4:**
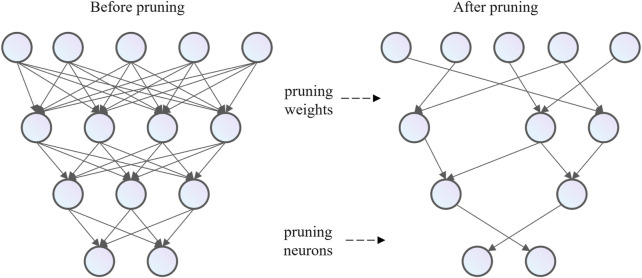
The structure of a neural network after pruning.

Model pruning and compression methods involve the removal of non-essential components from over-parameterized deep models. Pruning techniques are primarily categorized into weight pruning and unit pruning, depending on the level of pruning applied. Weight pruning, also referred to as unstructured pruning, compresses the network by eliminating insignificant neural connections, while unit pruning, also known as structured pruning, reduces the model size by removing neurons or convolutional filters. The central concept of neural network pruning is to eliminate parts deemed unimportant, redundant, or unnecessary based on their significance, thus simplifying the model without causing significant degradation in performance. As shown in Figure 4, unstructured weight pruning removes unnecessary, low-weight connections between layers in the neural network, while structured unit pruning eliminates all weight connections associated with specific neurons.

#### 3.2.1 Unstructured pruning

Researchers have proposed several weight-based pruning methods to remove unimportant weights. Han et al. (2015) introduced a pruning approach that eliminates weights whose absolute values fall below a predefined threshold, calculated as the product of a prime number and the standard deviation of the weights in a given layer. To enhance the accuracy of the pruned model, the network is reinitialized using the pre-pruning parameters and retrained. While the framework of Han et al. (2015) garnered significant early attention as a canonical pruning method (Liu et al., 2019), its irregular network structure requires specialized software or hardware accelerators, making it incompatible with standard libraries.

In Mocanu et al. (2018), the authors inspired by the sparsity of biological neural networks, argued that artificial neural networks should not be fully connected. They proposed replacing fully connected layers with a sparse topological structure, specifically an Erdos-Renyi random graph, which significantly reduces the number of training parameters without compromising predictive accuracy. During training, the smallest absolute weight connections are proportionally removed while new connections are added in equal measure. This approach identifies a sparse network structure but increases training costs. Additionally, the random connectivity of the unstructured sparse model leads to poor cache locality and inefficient memory access, which severely limits inference acceleration (Wen et al., 2016).

Pruning large portions of a model’s parameters at once often causes sharp performance degradation, so pruning is generally performed iteratively. In Frankle and Carbin (2019), the authors found that over-parameterized networks contain a sparse subnetwork, which they termed the “winning ticket” in their Lottery Ticket Hypothesis. After the model converges (i.e., the training accuracy plateaus), connections with the smallest absolute values are pruned, and the remaining network is reinitialized using the original parameters, followed by retraining until convergence. This process is repeated until either the desired compression ratio is achieved or performance significantly drops. Since each pruning step requires retraining the model from scratch, this method is computationally expensive. Frankle et al. (2020) later discovered that pruning based on convergence using the initial parameters fails to perform well in deeper networks. They also noted that the model stabilizes into a sparse structure early in the training process when exposed to data augmentation. Thus, they proposed resetting pruned model parameters to the weights obtained during early training, which shortens convergence time. Various extensions of the Lottery Ticket Hypothesis have since been proposed to explore its generalizability to different network architectures (Chen et al., 2020; Chen et al., 2021; Girish et al., 2021; Da Cunha et al., 2022; Bai et al., 2022).

In Liu et al. (2019), the authors argued that the network’s connectivity structure is more important than the inherited weights. They found that even when a pruned model is randomly reinitialized, it can still achieve comparable accuracy if trained for a similar amount of time. Furthermore, they demonstrated that using the original model’s initialization values for retraining the pruned network, as proposed by Frankle et al. (2020), offers no substantial benefits over random initialization, given optimal learning rates.

To address the limitations of unstructured pruning, researchers have investigated group-based sparsity strategies. Wen et al. (2016) introduced Structured Sparsity Learning (SSL), which applies group sparsity regularization to CNNs, leveraging the sparsity across different layers to compress the model. Lebedev and Lempitsky (2016) used group sparsity regularization to drive parameters toward zero, effectively eliminating connections associated with zeroed parameters. Zhou H. et al. (2016) imposed sparsity constraints on weights during training to construct sparse deep neural networks. While these structured sparsity methods have proven successful, they remove connections irregularly, meaning that specialized libraries or sparse matrix operations are still required for efficient inference in practice.

Weight-based pruning methods face practical limitations, mainly due to the unstructured connections they create. Current acceleration libraries do not support efficient inference on unstructured networks, requiring custom solutions to achieve inference speedups.

#### 3.2.2 Structured pruning

Structured pruning refers to the removal of a neuron along with all its input and output connections. Unlike unstructured pruning, structured pruning does not create sparse matrices. However, since structured pruning eliminates all connections to a neuron, the performance of the pruned network is often inferior to that achieved by weight-based pruning methods.

In He et al. (2014), the authors proposed a simple neuron-based pruning strategy that evaluates neuron importance by summing their output weights, pruning those deemed unimportant. They also introduced an entropy-based pruning approach, using a predefined threshold to assess the activation distribution of each neuron. As the accuracy of the pruned network decreases, fine-tuning is required to restore performance. Alqahtani et al. (2021) proposed a voting-based method, comparing neuron activations and assigning a score to evaluate their importance, thereby simplifying the model by removing less influential neurons. This approach identifies and removes unnecessary neurons during training, eliminating the need for pretraining or fine-tuning. Srinivas and Babu (2015) proposed pruning neurons by analyzing the similarity of weights within a layer; neurons with similar weights are pruned. Mariet and Sra (2016) introduced the DivNet model, which defines a probability measure over subsets of neurons and merges similar neurons based on their activation patterns. As with other pruning approaches, these methods lack ready-made libraries for implementation and require custom accelerators.

In convolutional networks, kernel-level pruning has also been extensively studied. These methods aim to assess the importance of intermediate convolutional kernels and prune those with the lowest importance scores. Li et al. (2017) proposed a simple method that ranks convolutional kernels by the sum of the absolute values of their weights and removes those with the smallest sums. Data-driven pruning methods have also been used to eliminate unimportant kernels. For instance, Polyak and Wolf (2015) designed a pruning method based on channel variance, using feature map activation variance to evaluate important filters and pruning unimportant kernels. Luo and Wu (2017) introduced a method that evaluates filter importance based on the entropy of their output channels, removing filters with the lowest entropy. Hu et al. (2016) proposed a pruning method that assesses filter importance by the average percentage of zero activations (APoZ) in output feature maps. They iteratively retrain the network using pre-pruning weights as initialization to achieve compression. Luo et al. (2019) developed the ThiNet method, which uses a greedy algorithm to select input channels that minimize reconstruction error, pruning channels that contribute more to the error. Liu et al. (2021) proposed an automatic channel-group pruning algorithm based on Fisher information, providing a unified metric to evaluate the importance of both individual and coupled channels.

Although Neuron-based pruning methods require specific support libraries, they generate structured weight matrices, which are more compatible with hardware accelerators for efficient performance. However, structured pruning generally performs worse than unstructured pruning, as it removes all the connections of a unit or cell, whereas unstructured pruning allows for the selective removal of individual connections across cells. This finer granularity often enables unstructured pruning to achieve better performance.

### 3.3 Low-rank decomposition

**FIGURE 5:**
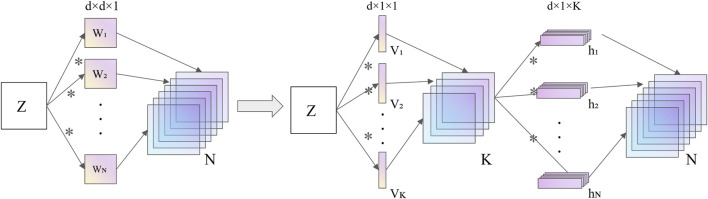
The core concept of low-rank decomposition.

Low-rank decomposition involves using matrix or tensor decomposition to identify key parameters in the model. A weight matrix is decomposed into the product of two smaller matrices, which perform functions similar to those of the original weight matrix. As shown in Figure 5, increasing the depth of the network and reducing the computational cost of individual convolutions can lead to a more lightweight model architecture while maintaining similar functionality.

In deep convolutional networks, convolution operations are the most computationally expensive, so compressing convolutional layers can significantly enhance both the acceleration ratio and the compression rate. A convolutional layer can be viewed as a 4D tensor (height, width, depth, and number of filters), but due to the significant redundancy within this structure, tensor decomposition has been applied as a model compression technique to reduce parameter redundancy.

Low-rank decomposition is used for model compression and acceleration, providing further speedup and resulting in smaller CNN models. Perona (1995) demonstrated that any convolutional kernel with rank R can be represented as a linear combination of R separable convolutional kernels. Building on this result, Rigamonti et al. (2013) proposed replacing high-dimensional convolutional kernels with a set of 1D kernels, using linear combinations to separate the original kernels, which achieves both compression and acceleration. This method approximates the original convolutional kernel with a combination of low-dimensional kernels and finds the optimal low-dimensional kernels by optimizing the distances between them. Jaderberg et al. (2014) extended this approach by replacing the original kernels with multiple low-rank convolutional kernels, compressing parameters and accelerating the model. Specifically, their method substitutes a single high-dimensional kernel with two layers of low-dimensional kernels, reducing computational costs through increased depth. For example, a 7×7 kernel is replaced by two kernels of 7×1 and 1×7 , reducing the parameter count from 49 to 14, achieving significant compression. Notably, while Rigamonti et al. (2013) optimized the distance between kernels to find the optimal solution, Jaderberg et al. (2014) focused on optimizing the feature maps generated by the kernels. Although both methods share common goals, the approach of Rigamonti et al. (2013) does not require data correction, while the method of Jaderberg et al. (2014) does.

Denil et al. (2013) investigated the redundancy in deep neural network parameters, hypothesizing that trained parameters can be predicted from a subset of other parameters. They introduced a compression technique that stores only a portion of the parameters and reconstructs the rest using a linear prediction model. Sainath et al. (2013) found that most of the parameters reside in the final fully connected layer, and low-rank decomposition of this layer can significantly reduce the number of parameters. Lu et al. (2017) proposed an automated method for designing compact multitask deep learning architectures. This method starts with a shallow network and expands it progressively during training, iterating to generate a tree-like deep network structure. This approach reduces a large number of redundant parameters, achieving effective compression of deep models.

Low-rank decomposition methods are indeed powerful, but they are not more widely applied due to a few key limitations. These methods often require a high computational cost during the decomposition process, which can be prohibitive for large models. Additionally, while they can reduce the parameter count, they may not always provide the same level of performance improvement as other methods, especially when applied to models with complex or highly nonlinear structures. Furthermore, low-rank methods can struggle with maintaining model accuracy after compression, particularly when fine-tuning is not sufficiently handled.

### 3.4 Knowledge distillation

**FIGURE 6:**
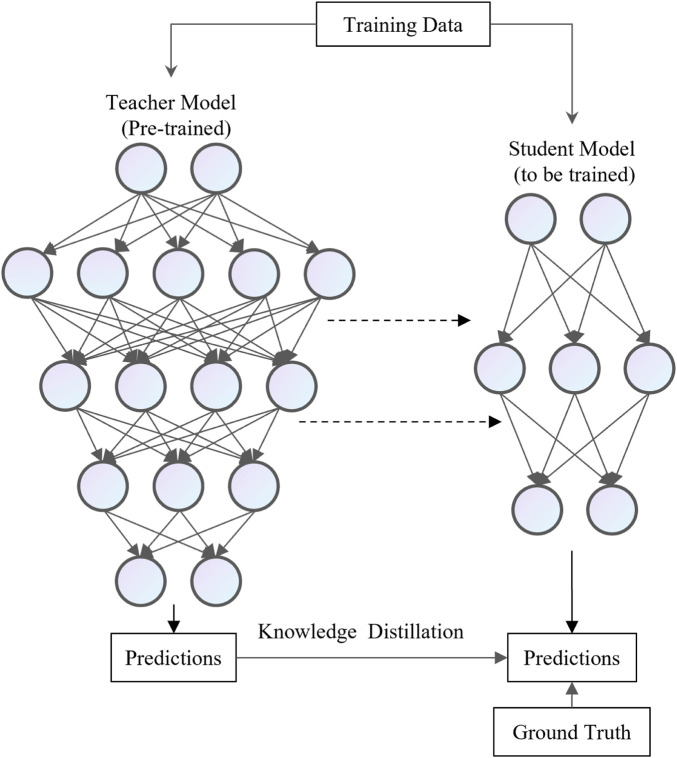
The process of model distillation.

Knowledge Distillation (KD) Hinton et al. (2015) is a valuable machine learning technique designed to enhance model performance and generalization. It works by distilling knowledge from a more complex model (called the teacher model) into a simpler model (called the student model). The fundamental concept behind KD is to transform the comprehensive knowledge of the teacher model into a more concise and efficient form, enabling the student model to replicate as much of the teacher model’s representational capacity as possible.As shown in Figure 6, distillation methods can be categorized into two types: black-box distillation and white-box distillation. In black-box distillation, only the predictions of the teacher model are accessible, while its parameters remain unavailable. In contrast, white-box distillation allows access to both the teacher model’s predictions and its parameters.

The white-box distillation method enables the student model to gain deeper insights into the teacher model’s internal structure and knowledge representation, often resulting in greater performance improvements. For example, Gu et al. (2024) proposed a knowledge distillation method to distill large language models (LLMs) into smaller models. They replaced the standard KD method’s forward Kullback-Leibler divergence (KLD) objective with a reverse KLD objective to prevent the student model from overestimating low-probability regions of the teacher’s distribution. They also developed an efficient optimization method to train this objective. These student models, named MINILLM, showed in experiments that they generated more accurate responses, improved overall quality, exhibited less bias, had better calibration, and outperformed baseline methods in long-text generation within an instruction-following setting.

However, existing KD methods for autoregressive sequence models face a distribution mismatch between the output sequences seen during training and those generated by the student model during inference. To resolve this, Agarwal et al. (2024) introduced Generalized Knowledge Distillation (GKD). GKD does not depend solely on a fixed set of output sequences but instead trains the student model by incorporating feedback from the teacher model on the student-generated sequences. Unlike traditional supervised KD methods, GKD also allows the flexibility to use alternative loss functions when the student model fails to replicate the teacher model’s distribution. Additionally, GKD facilitates the seamless integration of distillation with reinforcement learning (RL) fine-tuning for language models. Experiments confirmed the effectiveness of GKD in distilling autoregressive T5 models, including task-specific distillation for summarization, translation, and inference tasks, as well as task-agnostic distillation for instruction tuning.

As large language models (LLMs) continue to grow in size, there is a need for compression methods to reduce model size while maintaining their generalization ability and zero-shot prompting capabilities. To achieve task-agnostic, zero-shot distillation of LLMs without task-specific fine-tuning data, Jha et al. (2024) proposed initializing a truncated model using a subset of layers extracted from a larger model and then training it on pre-training data using a language modeling objective. Experimental results demonstrated that a simple layer-wise pruning method, combined with continued language model pretraining, can match or even surpass three existing state-of-the-art baselines, while improving computational efficiency by 1.5 times.

While distillation can indeed save time by speeding up inference, there are several other challenges to consider. One significant issue is the time-intensive nature of the process, particularly when working with large-scale teacher models, which require substantial computational resources. Additionally, the performance of the distilled student model is heavily dependent on the quality of the teacher model; an inadequate or suboptimal teacher can hinder the student’s learning. Another challenge is the potential loss of fine-grained knowledge during distillation, which may degrade the student model’s performance on specialized tasks. Furthermore, if the teacher and student models operate in different domains or are trained on distinct datasets, the distillation process may struggle to capture domain-specific nuances, limiting the student’s effectiveness. These factors emphasize the need for thoughtful design to address these difficulties.

## 4 Future research directions

Model compression technology, as one of the mainstream techniques for deploying models with large parameter counts to resource-constrained devices, has rapidly advanced in response to the increasing demands of various industries. However, there remains substantial potential for improvement in the practical deployment of current model compression methods. For instance, unstructured pruning still requires the development of specialized libraries for hardware support to enable acceleration. Based on the prior discussion of compression methods, this paper outlines several key research areas that warrant further attention and exploration.

### 4.1 Data-free compression methods

Current model compression techniques often rely on retraining or calibration to restore model accuracy to varying degrees. For example, pruning methods are highly dependent on retraining to recover accuracy, which requires significant GPU time. Even post-training quantization typically demands a calibration dataset to maintain accuracy. However, in scenarios where data privacy is critical, obtaining labeled calibration datasets can be challenging, and retraining or calibration lengthens the training process. Data-free compression methods, which compress models by analyzing the distribution of model weight parameters after training without requiring additional data for calibration, offer broader practical relevance. As such, research into data-free compression techniques represents a key future research direction.

### 4.2 Adaptive model compression methods

Neural architecture search (NAS) technology seeks to optimize model structure via feedback-driven processes, effectively using computational power to replace human effort in hyperparameter tuning. This approach allows for more precise model architectures and parameter settings. NAS has also made significant contributions to model compression. For instance, Cai et al. (2019) proposed ProxylessNAS, which not only reduces the floating-point operations of models but also lowers inference latency. However, research in this area remains limited, making adaptive model compression methods a promising avenue for future exploration.

### 4.3 Software-hardware co-design

The deep integration of multiple compression techniques to reduce model parameters is a dominant trend in model compression development. In particular, model pruning and parameter quantization have become standard in industry. In parameter quantization, mixed-precision methods—which combine floating-point and integer representations—have shown great success in retaining model capacity. However, while unstructured pruning enables finer compression, it is less suitable for hardware acceleration and requires the development of dedicated operator libraries. Similarly, mixed-precision quantization, though providing higher compression rates, also faces hardware acceleration challenges. Therefore, software-hardware co-design, which integrates both to optimize model acceleration, will be a critical research focus in the future.

## 5 Conclusion

Over-parameterized network models typically exhibit stronger learning capabilities and have shown outstanding performance across various domains. However, these models face significant challenges when deployed on edge devices due to the limited computational resources, which greatly hinders their practical application. Reducing the computational load of these models, without substantially sacrificing performance, is thus a key solution for enabling deployment on edge devices. This paper presents a comprehensive overview of model compression techniques, providing a detailed technical reference for deploying large-parameter models on edge devices. Finally, it is hoped that this paper will offer future researchers in deep model compression a thorough understanding of the field’s development and help address common challenges.
